# Vaginal Douching

**Published:** 1895-02-23

**Authors:** 


### EDITOR'S LETTER-BOX.

[Our correspondents are reminded that prolixity is a great bar to publication, and that brevity of style and conciseness of statement greatly facilitate early insertion.]

### VAGINAL DOUCHING.

Mr. Moffat Flynn, F.R.C.S.Ed., writes: My attention has been specially directed to this subject by the following case: A woman, suffering from chronic pelvic inflammation, was recommended to have hot vaginal douches containing Condy's fluid. They were given by a nurse of great intelligence, and with due care. The cervical canal was very free. At each douching the patient complained of pain, and felt faint on several occasions. Later she had an acute attack of pelvic inflammation, from which she slowly recovered. I entertained the idea that the douche might have been the cause of this. The best position to douche the vagina in is that adopted for the bi-manual examination; but as this is very irksome, the woman is usually put across the bed. I have observed that if the bed is the usual spring one in use in hospitals, that the shoulders are from four to six inches below the hips; that the uterus falls towards the abdominal wall; the os uteri being below the level of the vulvar opening of the vagina. The following takes place when women are douched in this position: the fluid accumulates in the vagina, the return fluid being merely an overflow. Some must go into the uterus, if the cervical canal is at all open, and from it into the peritoneal cavity, if a dilated tube exists. I expect the above case had a dilated tube. Such a train of events is very likely to happen in puerperal cases. When the douche is stopped, an ounce or two of fluid comes away, on the patient sitting up. I have adopted the following method: The patient is placed across the bed on her left side, the hips being well over the edge. Pillows are put under the left shoulder until it is at least eight inches higher than the corresponding hip. The perineum is pulled back by the fingers of one hand, and the vaginal pipe is introduced into the posterior cul de sac by those of the other. The can holding the fluid should be at least four feet above the patient's hips, this imparts a good force to the fluid, which spins round in the vagina and comes forcibly out, carrying secretions, &c., with it. When the douche is stopped the patient sits up in bed, when some fluid, less than half-an-ounce, comes away. By this method no fluid can enter the uterus, even if the cervical canal were well dilated. The cervix occupies the highest point of the vagina and there is a good fall towards the vulvar outlet. In the ordinary method the os uteri occupies the lowest part of the vagina and the fall is towards it.

